# ZIC1 Is Downregulated through Promoter Hypermethylation, and Functions as a Tumor Suppressor Gene in Colorectal Cancer

**DOI:** 10.1371/journal.pone.0016916

**Published:** 2011-02-15

**Authors:** Lihong Gan, Shujie Chen, Jing Zhong, Xian Wang, Emily K. Y. Lam, Xin Liu, Jianbin Zhang, Tianhua Zhou, Jun Yu, Jianmin Si, Liangjing Wang, Hongchuan Jin

**Affiliations:** 1 Department of Gastroenterology, Second Affiliated Hospital, School of Medicine, Zhejiang University, Hangzhou, China; 2 Laboratory of Digestive Disease, Clinical Institution of Sir Run Run Shaw Hospital, Zhejiang University, Hangzhou, China; 3 Key Laboratory of Biotherapy of Zheijang Province, Biomedical Research Center, Sir Run Run Shaw Hospital, School of Medicine, Zhejiang University, Hangzhou, China; 4 Department of Medicine and Therapeutics, Institute of Digestive Disease, Prince of Wales Hospital, The Chinese University of Hong Kong, Shatin, Hong Kong, SAR China; 5 Department of Cell Biology and Program in Molecular Cell Biology, School of Medicine Zhejiang University, Hangzhou, China; Florida International University, United States of America

## Abstract

The transcription factor, Zinc finger of the cerebellum (ZIC1), plays a crucial role in vertebrate development. Recently, ZIC1 has also been found to participate in the progression of human cancers, including medulloblastomas, endometrial cancers, and mesenchymal neoplasms. However, the function of ZIC1 in colon cancer progression has not been defined. In this study, we demonstrate ZIC1 to be silenced or significantly downregulated in colon cancer cell lines. These effects were reversed by demethylation treatment with 5-aza-2′-deoxycytidine (Aza). ZIC1 expression is also significantly downregulated in primary colorectal cancer tissues relative to adjacent non-tumor tissues (*p* = 0.0001). Furthermore, methylation of ZIC1 gene promoter is frequently detected in primary tumor tissues (85%, 34/40), but not in adjacent non-tumor tissues. Ectopic expression of ZIC1 suppresses cell proliferation and induces apoptosis, which is associated with MAPK and PI_3_K/Akt pathways, as well as the Bcl-xl/Bad/Caspase3 cascade. To identify target candidates of ZIC1, we employed cDNA microarray and found that 337 genes are downregulated and 95 genes upregulated by ectopic expression of ZIC1, which were verified by 10 selected gene expressions by qRT-PCR. Taken together, our results suggest that ZIC1 may potentially function as a tumor suppressor gene, which is downregulated through promoter hypermethylation in colorectal cancers.

## Introduction

Colorectal cancer (CRC) is the third most common cancer, as well as the third leading cause of cancer deaths worldwide [1]. The sequential accumulation of genetic and epigenetic alterations leads to the transformation of normal colonic epithelium to colorectal cancer [2]. These epigenetic changes, including promoter DNA methylation and histone modifications, can induce the inactivation of tumor suppressor genes (TSGs) [3]–[5]. DNA regions enriched with CpG dinucleotides, called CpG islands, can become hypermethylated in cancer cells and result in the silencing of TSGs [5]. A subset of CRCs display methylation of multiple genes, termed the CpG island methylator phenotype (CIMP) [2], [5]. We and others have previously found that growing numbers of TSGs including APC, CDKN2A/p16, UCHL1, and TBX5 are frequently silenced through promoter hypermethylation in CRCs [2], [6]–[9]. These events can occur in early stage of CRCs [9], [10], which highlights the importance of promoter hypermethylation in the tumorigenesis of CRCs.

Located at chromosome 3q24, ZIC1 encodes a C_2_H_2_-type zinc finger transcription factor that plays a critical role in the development of the neural crest and the cerebellum in vertebrates [11]–[15]. As zinc finger transcription factors, ZIC family proteins can bind to the GC-rich sequence in target genes [15]. Despite its role in neural development, ZIC1 was also found to participate in the progression of human cancers, such as medulloblastoma, endometrial cancers, and mesenchymal neoplasms [16]–[18]. We have identified ZIC1 as a novel candidate TSG in gastric cancer [19]. To support its role in cancer, ZIC1 can function as a repressor of the downstream target of sonic hedgehog (Shh), BMP (bone morphogenetic protein), and as well as play a role in Notch signaling pathway during neural tube development [14], [15]. However, the biological significance of DNA methylation, and the molecular mechanism underlying ZIC1 functioning as a TSG in CRCs remain unknown. Here, we report that ZIC1 promoter is frequently methylated in CRCs tissues and colon cancer cell lines. Ectopic expression of ZIC1 leads to cell growth inhibition, and alter the expression of potential target genes that may play important roles in colorectal carcinogenesis. Our results suggest that ZIC1 may potentially function as a novel functional tumor suppressor in CRCs.

## Results

### Transcriptional silencing of ZIC1 is associated with its promoter hypermethylation in colon cancer cells

**Figure 1:**
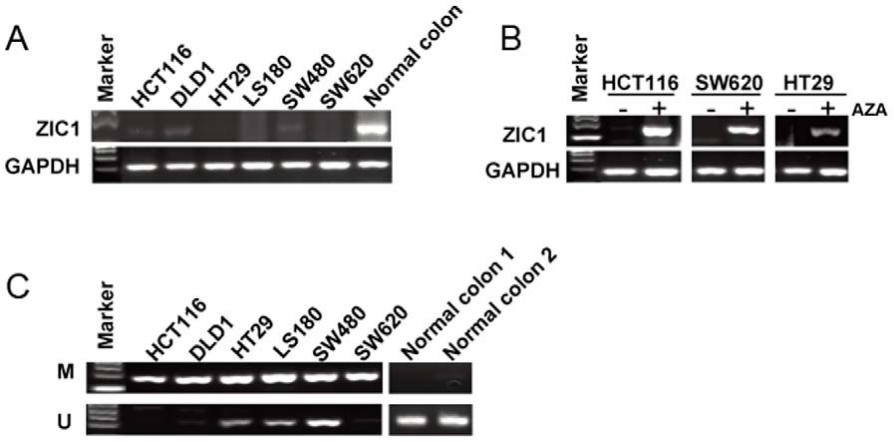
Promoter methylation contributes to ZIC1 downregulation in colon cancer cell lines. (**A**) The expression of ZIC1 is silenced or downregulated in colon cancer cell lines, when compared with normal colon tissue by RT-PCR. GAPDH was used as the internal control. **Normal colon**: Normal colon tissues. (**B**) ZIC1 expression is restored in colon cancer cells (HCT116, HT29 and SW620) after treatment with demethylation agent 5-Aza by RT-PCR. **AZA**: 5-aza-2′-deoxycytidine. (**C**) The methylation status of ZIC1 CpG promoter is detected by methylation-specific PCR (MSP) in colon cancer cell lines. **M**: methylated; **U**: unmethylated.

To determine whether ZIC1 is silenced by promoter hypermethylation in colon cancer, we examined the expression of ZIC1 mRNA in six colon cancer cell lines. Semi-quantitative RT-PCR showed that ZIC1 transcript was silenced or downregulated in all of colon cancer cell lines when compared to normal colon tissue (**Figure 1A**). The demethylation treatment by Aza dramatically restored the expression of ZIC1 mRNA in a subset of colon cancer cells (HCT116, HT29 and SW620) (**Figure 1B**), implicating that DNA methylation may be involved in the regulation of ZIC1 expression. Furthermore, we employed methylation specific PCR (MSP) and found that three colon cancer cell lines (HCT116, DLD1 and SW620) were detected with full methylation. The other three cell lines (HT29, LS180 and SW480) were found with partial methylation. No methylation was detected in the normal colon tissues (**Figure 1C**). Thus, these results indicate that transcriptional silence of ZIC1 in colon cancer cell lines may be mediated by DNA promoter hypermethylation.

### Downregulation and promoter hypermethylation of ZIC1 in primary colorectal tumors

**Figure 2:**
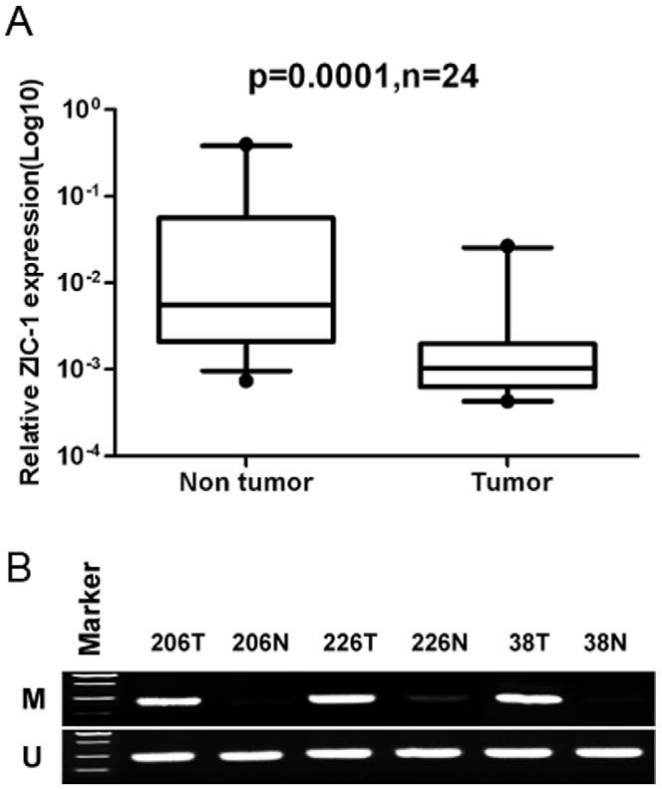
Downregulation of ZIC1 expression and frequent hypermethylation of ZIC1 promoter in primary colorectal tumor tissues. (**A**) ZIC1 mRNA expression was determined by quantitative real-time PCR in twenty four pairs of primary colorectal tumor and according adjacent non-tumor tissues. The data was analyzed by Wilcoxon matched pairs test. (**B**) The methylation status of ZIC1 promoter in primary colorectal carcinoma and adjacent non-tumor tissues was determined by MSP. Representative image are shown. **T**: tumor tissues; **N**: adjacent non-tumor tissues; **M**: methylated; **U**: unmethylated.

To further investigate the relationship between ZIC1 expression and promoter hypermethylation, we analyzed ZIC1 mRNA expression and CpG site methylation status in primary colorectal tumor and adjacent non-tumor tissues by qRT-PCR and MSP, respectively. The level of ZIC1 mRNA was significantly decreased in most tumor tissues relative to adjacent non-tumor tissues (*p* = 0.0001, n = 24) (**Figure 2A**). Additionally, promoter methylation was detected in 85% (34/40) of colorectal tumor tissues, but not in adjacent non-tumor tissues by MSP analysis (the representative data shown in **Figure 2B**), implying a tumor-specific hypermethylation of the ZIC1 promoter in CRCs. However, we failed to find a significant correlation between ZIC1 promoter methylation and clinical characteristics, such as age, gender, tumor differentiation, and TNM stage (**Table 1**).

**Table 1:** Clinicopathological features of ZIC1 methylation in colorectal cancer.

Characteristics	Methylated (n)	Unmethylated (n)	P-value
Age, years(±SD)	60.6±13.1	60.2±5.1	0.939
Gender			0.489
Male	19	4	
Female	15	2	
Differentiation			0.398
Well	21	3	
Morderate	12	2	
Poor	1	1	
TNM Stage			0.315
I	3	2	
II	14	3	
III	16	1	
IV	1	0	

### ZIC1 inhibits the proliferation of colon cancer cells

**Figure 3:**
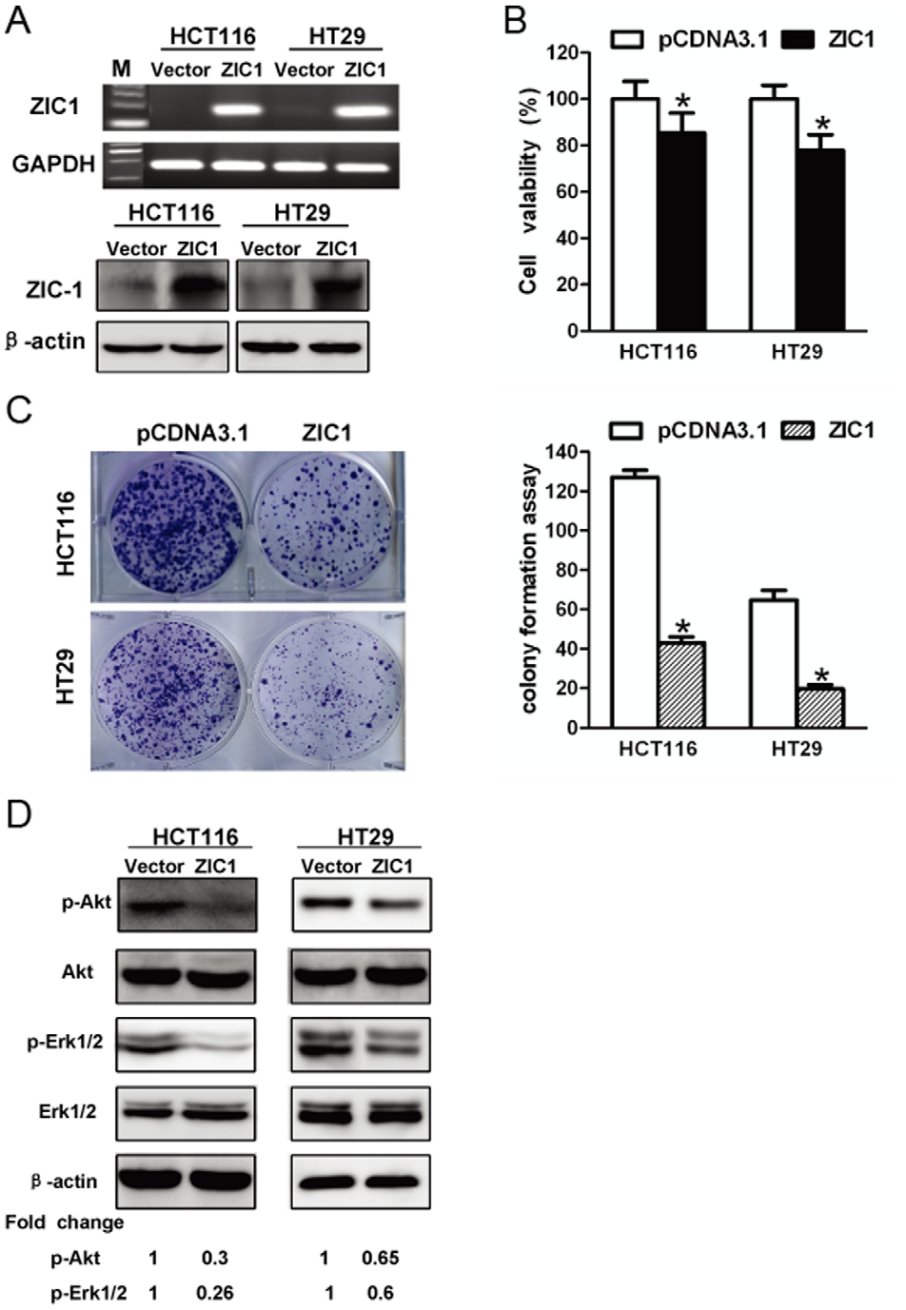
ZIC1 inhibits the proliferation of colon cancer cell and regulates MAPK, PI_3_K/Akt pathways. (**A**) Re-expression of ZIC in stable transfectants in colon cancer cells (HCT116 and HT29) was confirmed by RT-PCR (upper lane) and western blot (low lane), GAPDH and β-actin used as internal control. (**B**) Cell viability assay after ZIC1 re-expression in colon cell lines (HCT116 and HT29). The number of viable cells was measured by MTS assay after transiently transfected with pCDNA3.1-ZIC1 or pCDNA3.1 vector. The assay was performed in triplicate. The asterisk indicates statistical significance (*p*<0.05). (**C**) The effect of cell proliferation inhibition by ectopic expression of ZIC1 was determined by colony formation assay in colon cancer cells. The quantitative analysis of colony numbers formed in pCDNA3.1-ZIC1 or pCDNA3.1 vector transfectants are shown in right bar diagram in three individual experiments in HCT116 and HT29 cells. The asterisk indicates statistical significance (*p*<0.05). (**D**) The expression phos-Akt and phos-Erk1/2, as well as the total of Akt and Erk1/2 were analyzed by western blot. Band densities were quantified and protein levels (p-Akt, p-Erk1/2) were normalized to β-actin. Densitometry values (ZIC1 transfectants) are expressed as fold change compared with vector transfectants values normalized to 1.

To explore the effect of ZIC1 on cell proliferation in colon cancer, we performed cell viability and colony formation assays in CRC cell lines. First, the transfection efficiency of our ZIC1 construct was confirmed by RT-PCR and western blot in tumor cell lines (HCT116 and HT29) (**Figure 3A**). Next, we evaluated the suppressive effect of ZIC1 overexpression on cell proliferation by cell viability assay. As shown in **Figure 3B**, ectopic expression of ZIC1 significantly inhibited cell viability in HCT116 and HT29 cells (*p*<0.05). We also observed that the number of surviving colonies formed on the plates was significantly reduced when compared with the control vector transfectants (*p*<0.01) (**Figure 3C**). In addition, we revealed that ectopic expression of ZIC1 inhibited the phosphorylation of Erk1/2 and Akt kinases (**Figure 3D**), two key cell proliferation pathway regulators. These results confirmed the suppressive effect of ZIC1 on cell proliferation.

### Ectopic expression of ZIC1 induces cell apoptosis

**Figure 4:**
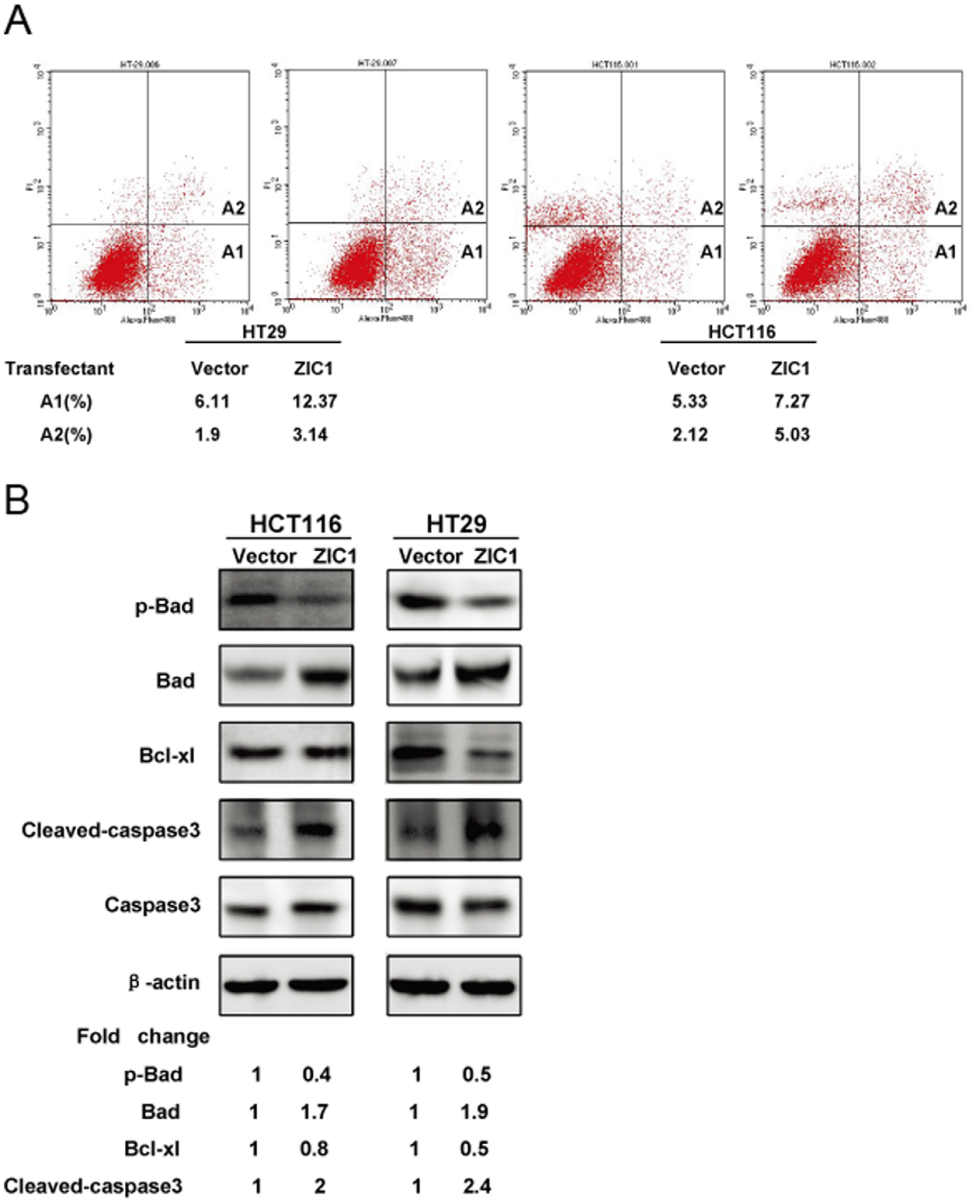
Ectopic expression of ZIC1 induces cell apoptosis and modulates Bcl-xl/Bad/Caspase3 cascade. (**A**) Cell apoptosis was detected by the Annuexin V-PI flowcytometry assay. The representative figures are shown after transiently transfected with ZIC1 or control vector after 48 hours in HT29 and HCT116 cells. Region **A1** indicates early apoptotic cells, **A2** shows late apoptotic cells. (**B**) Western blot analysis of apoptotic regulated proteins. The expression of phospho-Bad and Bad, Bcl-xl, Cleaved-caspase3, and Caspase3 were detected after transected with ZIC1 or control vector in colon cancer cell lines (HT29 and HCT116). Band densities were quantified and protein levels (p-Bad, Bad, Bcl-xl, and Cleaved-caspase3) were normalized to β-actin. Densitometry values (ZIC1 transfectants) are expressed as fold change compared with vector transfectants values normalized to 1.

To explore the mechanisms underlying the inhibition of cell proliferation by ectopic expression of ZIC1, we analyzed cell apoptosis and cell cycle by flow cytometry assay. As shown in **Figure 4A**, the cells transfected with ZIC1 induced cell apoptosis. In addition, our results showed that re-expression of ZIC1 led to the activating of apoptosis-related cascades, including cleavage of caspase3, downregulation of Bcl-xl, and Bad dephosporylation (**Figure 4B**). These results indicate that the induction of cell apoptosis through overexpression of ZIC1 is mediated by the Bcl-xl/Bad/Caspase3 cascade. However, re-expression of ZIC1 did not affect cell cycle progression (data not shown).

### Gene expression profile changes by ectopic exogenous ZIC1 expression

**Figure 5:**
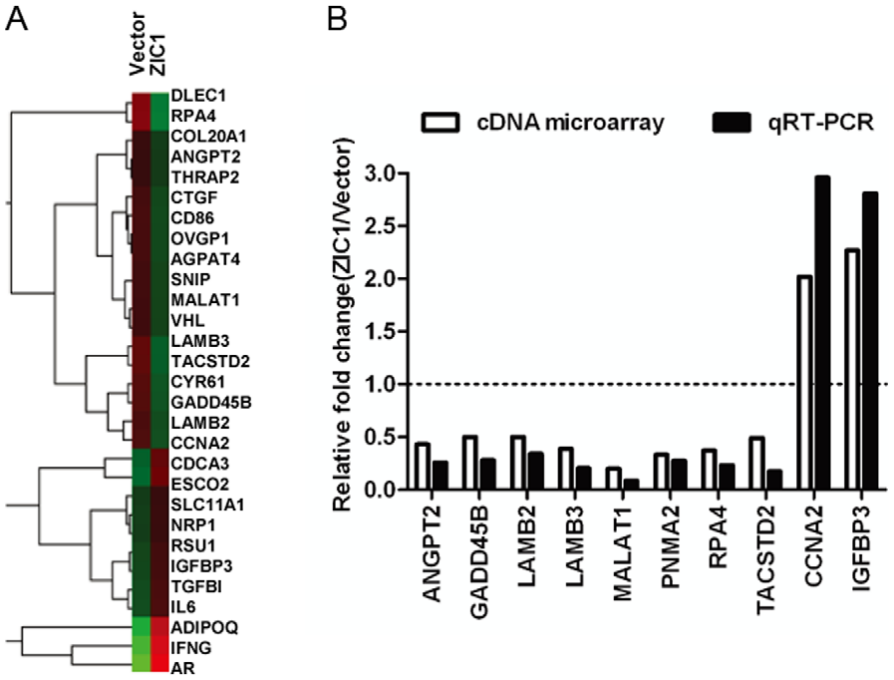
The validation of ZIC1 regulating target genes. (**A**) The differential expression of gene profiles in ZIC1 or control vector stable transfectants was visualized using Java Treeview in HCT116 cell lines. Representative genes over two-fold change are indicated on the right side of this image. (**B**) Genes transcript quantity of 10 selected genes was measured by qRT-PCR and calculated using the value of 2^−△△CT^. Relative gene expression in ZIC1 transfectants was compared with empty control vector, which was normalized as a reference value of 1.0. White bars indicate the result of microarray analysis, and black bars representative of qRT-PCR data in HCT116 cell line.

To search for target genes of ZIC1 in colon cancer cells, we utilized cDNA microarray to analyze gene expression profile changes induced by ectopic expression of ZIC1. This analysis revealed that 337 genes were downregulated and 95 genes were upregulated by exogenic expression of ZIC1 when using a 2-fold change of expression as threshold (representative genes shown in **Figure 5A**). As shown in **Table 2**, many of these genes have been reported to play important roles in cell proliferation and apoptosis. To confirm the expression pattern observed in the microarray, we validated the expression of 10 selected genes in colon cancer cells transfected with ZIC1 by qRT-PCR. The results showed that *CCNA2* (cylin A2) and *IGFBP3* (insulin-like growth factor binding protein 3) were significantly upregulated (>2 fold change), whereas *ANGPT2* (angiopoietin 2), *GADD45B* (growth arrest and DNA-damage-inducible, beta), *LAMB2* (laminin, beta 2), *LAMB3* (laminin, beta 3), *MALAT1* (metastasis associated lung adenocarcinoma transcript 1), *PNMA2* (paraneoplastic antigen MA2), *RPA4* (replication protein A4), and *TACSTD2* (tumor-associated calcium signal transducer 2) (<−2 fold change) were downregulated by overexpression of ZIC1 in HCT116 and HT29 cells (**Figure 5B** **and Table S1**). These data were concordant with that obtained from the microarray analysis.

**Table 2:** Representative gene expression profile in ZIC1 transfectants compared with empty vector control (fold change) by cDNA microarray in HCT116 cells.

Gene Symbol	Gene Description	GenbankAccession	FoldChange (*)	Gene Function
			Down-Regulation	cell proliferation, apoptosis, migration, adhesion, and angiogenesis
ANGPT2	angiopoietin 2	NM_001147	0.43	angiogenesis
AOC3	amine oxidase, copper containing 3	NM_003734	0.48	cell adhesion
AVIL	advillin	NM_006576	0.47	cell migration
COL20A1	collagen, type XX, alpha 1	NM_020882	0.37	cell adhesion
CTGF	connective tissue growth factor	NM_001901	0.43	cell proliferation/angiogenesis
CYR61	cysteine-rich, angiogenic inducer, 61	NM_001554	0.42	cell proliferation/cell adhesion
DLEC1	deleted in lung and esophageal cancer 1	NM_007335	0.45	cell proliferation
EFNB3	ephrin-B3	NM_001406	0.47	cell differentiation
GADD45B	growth arrest and DNA-damage-inducible, beta	NM_015675	0.5	cell proliferation/cell apoptosis
KRT14	keratin 14	NM_000526	0.48	epithelial cell differentiation
LAMB2	laminin, beta 2 (laminin S)	NM_002292	0.5	cell adhesion/cell migration
LAMB3	laminin, beta 3	NM_001017402	0.39	cell adhesion/cell migration
MALAT1	metastasis associated lung adenocarcinoma transcript	NR_002819	0.2	cell proliferation/metastasis
MLXIPL	MLX interacting protein-like	NM_032951	0.5	cell proliferation
OSM	oncostatin M	NM_020530	0.49	cell proliferation
PNMA2	paraneoplastic antigen MA2	NM_007257	0.33	cell proliferation
PTPRH	protein tyrosine phosphatase, receptor type, H	NM_002842	0.44	cell apoptosis
RPA4	replication protein A4, 34 kDa	NM_013347	0.37	cell proliferation
SNIP	Smad nuclear interacting protein 1	NM_025248	0.46	cell migration
TACSTD2	tumor-associated calcium signal transducer 2	NM_002353	0.49	cell proliferation

			Up-Regulation	cell proliferation, apoptosis, migration, adhesion, and angiogenesis
CCNA2	cyclin A2	NM_001237	2.02	cell proliferation
CDCA3	cell division cycle associated 3	NM_031299	2.07	cell proliferation
ESCO2	establishment of cohesion 1 homolog 2	NM_001017420	2.43	cell proliferation
IBSP	integrin-binding sialoprotein	NM_004967	2.11	cell adhesion
IGFBP3	insulin-like growth factor binding protein 3	NM_001013398	2.27	cell proliferation/cell apoptosis
NRP1	neuropilin 1	NM_003873	2.47	cell adhesion/angiogenesis
ORC1L	origin recognition complex, subunit 1-like	NM_004153	2.34	cell proliferation
PLEKHK1	rhotekin 2	BC025765	2.03	cell proliferation
SPP1	secreted phosphoprotein 1	NM_000582	2.49	cell adhesion
TGFBI	transforming growth factor, beta-induced, 68 kDa	NM_000358	2.28	cell adhesion/cell proliferation

*Fold Change: ZIC1 versus control vector.

## Discussion

In the present study, we found that ZIC1 was silenced or downregulated in colon cancer cell lines, as well as in primary tumor tissues relative to adjacent non-tumor tissues (*p*<0.05). Furthermore, our observations identify that promoter DNA hypermethylation contributes to ZIC1 silencing or downregulation in CRCs. These results are consistent with our previous study of ZIC1 in gastric cancer [19], indicating that promoter CpG methlyation is the predominant mechanism for ZIC1 downregulation. However, we cannot exclude the appearance of other mechanisms in silencing of ZIC1, such as histone or nucleosome remodeling. For instance, ZIC1 expression failed to be restored after Aza treatment in LS180 and SW480 cell lines (data not shown). Recent studies illustrate that methylation of histone H_3_ at lysine 27 is linked to ZIC1 silencing in embryonic stem cells [18]. Chromatin immunoprecipitation assays show that the expression of ZIC1 is associated with histone H_3_ dimethylation at lysine 4 in desmoids and MEF cells [18].

Despite the illustration of the critical role of ZIC1 in vertebrate development [13], [15], functional characterization of ZIC1 in carcinogenesis remains largely unknown. Here, our results show that exogenous ZIC1 inhibits cell proliferation through p-Akt and p-Erk1/2 inactivation in colon cancer cells. PI_3_K/Akt and MAPK (Mitogen activated protein kinase) signaling pathways are well known to function as crucial components of cell proliferation in tumor cells [20], [21]. Akt and Erk1/2, once activated by phosphorylation, can function as key effectors of PI_3_K and MAPK signaling pathways to promote cell survival and proliferation [20]–[24]. Thus, ZIC1 may mediate cell proliferation through PI_3_K/Akt and MAPK pathways in colon cancer cells. In addition, we found that ectopic expression of ZIC1 can induce apoptosis of colon cancer cells. Among the wide spectrum of proteins and genes involved in apoptosis, members of the Bcl-2 family play a central role in this process [25], [26]. Caspase-3 has been identified as being a key mediator of apoptosis in mammalian cells, and the phosphorylation of Bad can block its apoptotic function [22], [25], [26]. In this regard, we found that the expression of cleaved-caspase3 and Bad were induced, while phospho-Bad and Bcl-xl were suppressed by re-expression of ZIC1. Our results suggest that induction of apoptosis by ZIC1 may be associated with Bcl-xl/Bad/Caspase3 cascade in colon cancer cells.

In an attempt to identify downstream targets of ZIC1 in CRCs, we analyzed the gene expression profiles of colon cancer cells with or without ZIC1 overexpression. The results revealed that 337 genes were downregulated and 95 genes were upregulated by ZIC1 (representative novel genes shown in **Table 2**). Many of these genes have been linked to cellular growth, apoptosis, adhesion, angiogenesis, and signal transduction in tumorigenesis [27]–[33]. For example, ZIC1 repressed the expression of *GADD45B*. *GADD45B* is induced by the activation of the p38/JNK (c-Jun N-terminal kinase) pathway [27], and an important mediator of NF-κB-JNK crosstalk and cell apoptosis [28]. JNK is another major downstream component of the MAPK cascades, and is associated with cell growth and cellular response to DNA damage [21], [23], [24]. In addition, we found that ZIC1 increased the expression of *RSU1* (Ras suppressor protein 1), which is reported to elevate the levels of p21^CIP^ CDK inhibitor, as well as inactivate Jun and Rho-dependent kinases under EGF stimulation [29]. With our finding of ZIC suppression of p-Erk1/2, we propose that ZIC1 can regulate MAPK pathways mediated by ERK and JNK kinases. Further study is required to illustrate the mechanisms by which ZIC1 regulates these potential pathways in cancer progression. Furthermore, we demonstrated that ZIC1 can suppress the expression of other novel genes (*TACSTD2*, *ANGPT2*, *LAMB2, LAMB3* and *MALAT1* etc.) related to tumor angiogenesis and metastasis. *TACSTD2* has been found associated with tumor aggressiveness and poor prognosis in epithelial cell tumors, including colon and stomach cancer [30], [31]. *ANGPT2* is emerging as a key regulator of vascular remodeling during tumor angiogenesis [32], [33].

As zinc finger transcription factors, the ZIC family of proteins can bind to GC-rich sequences in target genes [13], [15]. ZIC1 may regulate target genes in both sequence-specific and sequence-independent manners [15]. Depending on its interaction partners, ZIC proteins can activate or suppress the transcription of target genes. As expected, we observed that ZIC1 regulated the expression of important transcription factors such as *RPS2*, *NTF3*, *PRDM16*, *KLF15*, *SHC2* and *FOXJ1* (**Table S2**). ZIC1 has been shown to counteract with Gli (glioma-associated oncogene homolog 1), which functions as downstream of sonic hedgehog (Shh) signaling pathway and participate in the progression of colon cancer [34]–[36]. Meanwhile, numerous of downstream targets of ZIC1 including Notch, Cyclin D1, and Wnt3a have been reviewed in neural development and animal models [15], [37]. These genes are well known to play vital roles in cancer development. The study of ZIC1 target genes may provide further insight into the possible mechanisms of ZIC1 serving as a tumor suppressor in CRCs.

In summary, we revealed that a novel tumor suppressor gene ZIC1 was inactivated through promoter methylation in colon cancer cells. ZIC1 was also downregulated and frequently hypermethylated in primary colorectal cancer tissues. ZIC1 inhibits cell proliferation through suppression of PI_3_k and MAPK pathways, induction of cell apoptosis through the Bcl-xl/Bad/Caspase3 cascade, regulation of downstream targets and pathways implicated in colorectal carcinogenesis.

## Materials and Methods

### Cell culture and tissue specimens

The human colon cancer cell lines (HCT116, HT29, DLD1, LS180, SW480 and SW620) were obtained from Riken Gene Bank (Japan) and American Type Culture Collection (ATCC, USA). HCT116 cell line was cultured in McCoy's 5A medium (Invitrogen, USA) supplemented with 10% fetal bovine serum, all other cell lines were cultured in DMEM medium (Invitrogen, USA) supplemented with 10% fetal bovine serum. All cell lines incubated at 5% CO_2_, 37°C and 95% humidity.

Forty surgical resected colorectal adenocarcinomas and adjacent non-tumor specimens were obtained from Sir Run Run Shaw Hospital, School of Medicine Zhejiang University. CRC was classified according to International Union Against Cancer Criteria and staged with the tumor-node-metastasis (TNM) system. Specimens were immediately frozen in liquid nitrogen and stored in −80°C until further processing. All patients provided informed written consent for obtaining the study specimens. The study protocol was approved by the Clinical Research Ethics Committee of Sir Run Run Shaw Hospital.

### Pharmacological DNA demethylation with 5-Aza-2′-Deoxycytidine

Cells were treated for 72 hours with 5 µM 5-*aza*-2′-*deoxycytidine* (Aza) (Sigma, St Louis, MO, USA), a well-used methyltransferase inhibitor. Aza was replenished every 24 hours. An equivalent concentration of the vehicle (DMSO) was used as the control.

### RT-PCR and quantitative PCR analysis

Total RNA (1 µg) was extracted using Trizol reagent (Invitrogen) following manufacturer's instruction, then reverse transcribed into cDNA with High-capacity cDNA Reverse Transcription kit (Applied Biosystems). RT-PCR was carried out for 35 cycles (95°C for 30 s, 55°C for 30 s, 72°C for 30 s). PCR product was electrophoresed in 2% agarose and stained with ethidium bromide. Quantitative real-time PCR (qRT-PCR) was performed with the SYBR Green Master Mix kit (Takara,Japan) in ABI 7500 PCR system. The relative gene transcript level was normalized to house keeping gene (GAPDH) using the 2^−ΔΔCt^ method. All primers sequences were listed in **Table S3**.

### Bisulfite treatment of DNA and methylation specific PCR (MSP)

Genomic DNA (1 µg) was bisulfite-treated with Zymo DNA modification Kit (Zymo Research, USA). Methylation status of ZIC1 was detected by methylation-specific PCR (MSP) using the bisulfite treated genomic DNA as the template. MSP was carried out for 40 cycles with annealing temperature at 60°C, as previously described [38], [39]. Methylation (M) primers were: F 5′-GGATTTTTTGTTTCGTAATC, R 5′-CCCGTTAACCACGTTAAACG, and Unmethylation (U) primers were: F 5′-GGGATTTTTTGTTTTGTAATT, R 5′-CCCATTAACCACATTAAACA.

### Cell transfection and cell viability assay

To construct a ZIC1 expression plasmid, the full-length ZIC1 open reading frame was cloned into mammalian expression vector pCDNA3.1 as previous described [19]. To generate stable transfection cells, HCT116 and HT29 cells were transfected with pCDNA3.1-ZIC1 or pCDNA3.1 vector using Lipofectamine 2000 (Invitrogen), and selected by G418 (400 µg/ml) for 14 days in a 12-well plate. The overexpression of ZIC1 was confirmed by RT-PCR and Western blot in the surviving colonies. Then these stable heterogeneous populations of cells were transferred into 6-well plate to continuous selection with G418 for further studies.

Cell viability was determined by 3-(4,5-dimethylthiazol-2-yl) -2-(4-sulfophenyl)-2H -tetrazolium (MTS) reagents (Promega, Madison, USA). Briefly, HCT116 and HT29 cells were cultured 24 hours in a 12-well plate and transiently transfected with pCDNA3.1-ZIC1 or pCDNA3.1. Then these cells were plated in 96-well (2000–4000 cells/well) for 48 hours. After incubation with CellTiter 96 Aqueous One Solution reagent for 1 hour, the absorbance was measured at 490 nm according to the instruction of MTS.

### Colony formation assay

HCT116 and HT29 cells were cultured in 12- well plate (1.0×10^5^ cells/well) for 24 hours and transfected with pCDNA3.1-ZIC1 or pCDNA3.1 vector. After 48 hours, the transfectants were re-plated in 6-well plate and cultured for 12–20 days in culture medium containing G418 (400 µg/ml). Surviving colonies were stained with Gentian Violer after methanol fixation and visible colonies (≥50 cells) were counted. The experiments were performed in triplicate.

### Cell apoptosis and cell-cycle analysis

Cell apoptosis assays were performed using the annexin V/PI kit (Invitrogen) by flow cytometry analysis (FCA). Briefly, transiently transfected cells (HCT116, HT29) were suspended in annexin-binding buffer, Alexa Fluor 488 annexin V and PI working solution were added in sequence. The stained cells were finally analyzed by flow FACScan flow cytometry (Becton Dickinson, USA) at 560 nm. Meanwhile, 2×10^5^ seeded cells were exposed to the ultraviolet to induce apoptosis as a positive control.

Cell cycle distribution was detected by the Cycletest Plus DNA Reagent kit (Becton Dickinson, USA). Briefly, transfected cells were harvested and washed in PBS, cellular DNA was stained with 125 µg/mL propidium iodide for 20 minutes at 4°C in the dark. The cells then were sorted by FACS Calibur and cell-cycle distribution was determined using the ModFit LT software (Phoenix, USA).

### Western blot analysis

Total proteins were extracted from stably transfected cells using RIPA lysis buffer. Lysates (20–60 µg) were resolved on SDS-PAGE gel and transferred to PVDF membranes (Millipore, Bedford, MA). The blots were probed with ZIC1 (1∶500; Abcam), Caspase3 (1∶500; Sigma-Aldrich), Bcl-xl (1∶500; Sigma), Bad (1∶500; Abnova), phospho-Bad (1∶1000; Cell Signaling), Akt (1∶500; Abcam), phospho-Akt (1∶2000;Cell Signaling), Erk1/2 (p44/42) (1∶1000; Cell Signaling), phospho-Erk1/2 (p44/42) (1∶2000;Cell Signaling) and β-actin (1∶2500, Multisciences Biotech) antibodies. The blots were developed using a chemiluminescence with Las-4000 Imaging System (Fujifilm, Japan).

### cDNA microarray analysis and validation

Microarray studies were filtered to identify those that profiled gene expression in ZIC1 or control vector stably transfected cell line (HCT116) based on Affymetrix platform. cDNA was transcribed into cRNA with aaUTP binding, which allow incorporation of fluorescent dye Cy3 (pCDNA3.1-ZIC1) or Cy5 (pCDNA3.1). Finally, labeled samples were hybridized to Agilent whole human genome containing 41,000 probes and transcripts. Duplicate experiments were carried out. We selected log_2_ ratio ≥1 or ≤−1 as the threshold for upregulation or downregulation of gene expression.

The candidate ZIC1 target genes were classified into different subgroups according to their biological functions (cell proliferation, migration, and angiogenesis, etc.). Ten representatives of target genes: *ANGPT2*, *CCNA2*, *GADD45B*, *IGFBP3*, *LAMB2*, *LAMB3*, *MALAT1*, *PNMA2*, *RPA4* and *TACSTD2* were verified with qRT-PCR in ZIC1 or control vector tranfectants in HCT116 and HT29 cells.

### Statistical analysis

Student's *t* and Wilcoxon matched pairs tests were performed to compare with two-independent data, while Chi-square or fisher exact test methods to analysis categorical variables. A *p*<0.05 was considered statistically significant.

## Supporting Information

Table S1 **Fold change (FC) of selected genes in ZIC1 transfecants were detected by cDNA microarray and qRT-PCR.** Fold change (FC): ZIC1 versus control vector.
(DOC)

Table S2 **Expression profile of representative gene associated with transcription regulator and signal transduction in ZIC1 transfectants compared with empty vector control (fold change) by cDNA microarray in HCT116 cells.** Fold change: ZIC1 versus control vector**.**
(DOC)

Table S3 **Primer sequences used for quantitative real-time PCR.**
(DOC)
