# Supplementary material for: ZIC1 Is Downregulated through Promoter Hypermethylation, and Functions as a Tumor Suppressor Gene in Colorectal Cancer
Source: PLoS One. 2011 Feb 15;6(2):e16916. doi: 10.1371/journal.pone.0016916 (PMC3039653; doi:10.1371/journal.pone.0016916)
Supplement: Table S1 — Fold change (FC) of selected genes in ZIC1 transfecants were detected by cDNA microarray and qRT-PCR. Fold change (FC): ZIC1 versus control vector. (DOC) [file pone.0016916.s001.doc]

**Table S1.**  Fold change (FC) of selected gene in ZIC1 transfecants detected by cDNA microarray and qRT-PCR (ZIC1 versus Vector control).

| **Gene Symbol** | **Gene Name** | **cDNAmicroarray**  **FC** | **qRT-PCR(HCT116)**  **FC** | **qRT-PCR(HT29)**  **FC** |
| --- | --- | --- | --- | --- |
| ANGPT2 | angiopoietin 2 | 0.43 | 0.26 | 0.05 |
| GADD45B | growth arrest and DNA-damage-inducible, beta | 0.50 | 0.28 | 0.36 |
| LAMB2 | laminin, beta 2 | 0.50 | 0.34 | 0.13 |
| LAMB3 | laminin, beta 3 | 0.39 | 0.21 | 0.23 |
| MALAT1 | metastasis associated lung adenocarcinoma transcript 1 | 0.2 | 0.09 | 0.19 |
| PNMA2 | paraneoplastic antigen MA2 | 0.33 | 0.28 | 0.30 |
| RPA4 | replication protein A4 | 0.37 | 0.23 | 0.36 |
| TACSTD2 | tumor-associated calcium signal transducer 2 | 0.49 | 0.17 | 0.25 |
| CCNA2 | cylin A2 | 2.02 | 2.96 | 1.84 |
| IGFBP3 | insulin-like growth factor binding protein 3 | 2.27 | 2.81 | 1.37 |

Fold Change(FC): ZIC1 versus control vector .
