# Supplementary material for: ZIC1 Is Downregulated through Promoter Hypermethylation, and Functions as a Tumor Suppressor Gene in Colorectal Cancer
Source: PLoS One. 2011 Feb 15;6(2):e16916. doi: 10.1371/journal.pone.0016916 (PMC3039653; doi:10.1371/journal.pone.0016916)
Supplement: Table S2 — Expression profile of representative gene associated with transcription regulator and signal transduction in ZIC1 transfectants compared with empty vector control (fold change) by cDNA microarray in HCT116 cells. Fold change: ZIC1 versus control vector. (DOC) [file pone.0016916.s002.doc]

**Table S2. Expression profile of representative gene associated with transcription regulator and signal transduction in ZIC1 transfectants compared with empty vector control (fold change) by cDNA microarray in HCT116 cells.**

| **Gene Symbol** | **Gene Description** | | **Genbank**  **Accession** | | **Fold**  **Change (*)** | | **Gene Function** | |  |
| --- | --- | --- | --- | --- | --- | --- | --- | --- | --- |
|  |  | |  | | **Down**  **-Regulation** | | **transcription regulator and signal transduction/pathway activity** | |  |
| CARD9 | caspase recruitment domain family, member 9 | | NM_052813 | | 0.49 | | regulation of NF-kB pathway | |  |
| CHRD | chordin | | NM_003741 | | 0.47 | | BMP signaling pathway | |  |
| EID3 | EP300 interacting inhibitor of differentiation 3 | | NM_001008394 | | 0.48 | | transcription regulator | |  |
| HEY1 | hairy/enhancer-of-split related with YRPW motif 1 | | NM_012258 | | 0.43 | | Notch signaling/regulation of transcription | |  |
| HSPA1A | heat shock 70kDa protein 1A | | NM_005345 | | 0.36 | | nucleotide binding | |  |
| HSPA6 | heat shock 70kDa protein 6 (HSP70B') | | NM_002155 | | 0.38 | | nucleotide binding | |  |
| IRX5 | iroquois homeobox 5 | | NM_005853 | | 0.47 | | transcription regulator/ cell cycle | |  |
| KLF15 | Kruppel-like factor 15 | | NM_014079 | | 0.47 | | transcriptional regulator | |  |
| MFNG | MFNG O-fucosylpeptide 3-beta- N-  acetylglucosaminyltransferase | | NM_002405 | | 0.46 | | Notch signaling | |  |
| MYOG | myogenin (myogenic factor 4) | | NM_002479 | | 0.37 | | transcription regulator | |  |
| MYOZ3 | myozenin 3 | | NM_133371 | | 0.46 | | modulation of calcineurin signaling | |  |
| PRDM16 | PR domain containing 16 | | NM_022114 | | 0.45 | | transcription regulator | |  |
| RHOBTB1 | Rho-related BTB domain containing 1 | | NM_014836 | | 0.50 | | signal transduction | |  |
| RNASE1 | ribonuclease, RNase A family, 1 (pancreatic) | | NM_198232 | | 0.37 | | nucleic acid binding | |  |
| SHC2 | | SHC (Src homology 2 domain containing) transforming protein 2 | | NM_012435 | | 0.49 | | Ras protein signal transduction | |
|  | |  | |  | | **Up**  **-Regulation** | | **transcription regulator and signal transduction/pathway activity** | |
| ARHGAP11A | | Rho GTPase activating protein 11A | | NM_014783 | | 2.10 | | signal transduction | |
| ATOH8 | | atonal homolog 8 (Drosophila) | | NM_032827 | | 2.30 | | transcription regulator | |
| FOXJ1 | | forkhead box J1 | | NM_001454 | | 2.99 | | transcription factor | |
| PDE4DIP | | phosphodiesterase 4D interacting protein | | AB042555 | | 2.02 | | cAMP-dependent pathway | |
| POU2F3 | | POU class 2 homeobox 3 | | NM_014352 | | 2.25 | | transcription regulator | |
| PRKAR1B | | protein kinase, cAMP-dependent, regulatory, type I, beta | | NM_002735 | | 2.08 | | cAMP signaling | |
| RSU1 | | Ras suppressor protein 1 | | NM_012425 | | 2.05 | | signal transduction | |
| SHCBP1 | | SHC SH2-domain binding protein 1 | | NM_024745 | | 2.27 | | SHC signal pathway | |

*****Fold Change: ZIC1 versus control vector .
