# Supplementary material for: ZIC1 Is Downregulated through Promoter Hypermethylation, and Functions as a Tumor Suppressor Gene in Colorectal Cancer
Source: PLoS One. 2011 Feb 15;6(2):e16916. doi: 10.1371/journal.pone.0016916 (PMC3039653; doi:10.1371/journal.pone.0016916)
Supplement: Table S3 — Primer sequences used for quantitative real-time PCR. (DOC) [file pone.0016916.s003.doc]

**Table S3. Primer sequences used for quantitative real-time PCR**

| Gene | Primer | Sequence | PCR product (bp) |
| --- | --- | --- | --- |
| ANGPT2 | Forward  Reverse | 5’ CCACGAATGGCATCTACACG 3’  5’ CCCAGCCAATATTCTCCTGA 3’ | 190 |
| CCNA2 | Forward  Reverse | 5’ TCCATGTCAGTGCTGAGAGGA 3’  5’ GAAGGTCCATGAGACAAGGC 3’ | 451 |
| GADD45B | Forward  Reverse | 5’ TCGGATTTTGCAATTTCTCC 3’  5’ GGATGAGCGTGAAGTGGATT | 302 |
| IGFBP3 | Forward  Reverse | 5’ GGCCATGACTGAGGAAAGGA 3’  5’ CCTGACTTTGCCAGACCTTCTT 3’ | 85 |
| LAMB2 | Forward  Reverse | 5’ CCCTGAGCCTGACAGACATAAATG 3’  5’ TGCTGAGGATGCTACCACCTTC 3’ | 231 |
| LAMB3 | Forward  Reverse | 5’ TCAGAGGAAGAGGGAGCAGTTTG 3’  5’ GGTCAGGCAACGAAGACATCTC 3’ | 251 |
| MALAT1 | Forward  Reverse | 5’ AAAGCAAGGTCTCCCCACAAG 3’  5’ GGTCTGTGCTAGATCAAAAGGCA 3’ | 71 |
| PNMA2 | Forward  Reverse | 5’ ACACTCGGGAACACATCAACAG 3’  5’ TGGGAAAATGCCTTCCACTCT 3’ | 93 |
| RPA4 | Forward  Reverse | 5’ ATAGGCGCTTTGACCAGCTA 3’  5’ ACGCTACCTGTGGACCAATC 3’ | 168 |
| TACSTD2 | Forward  Reverse | 5’ ACCCGAGGAGAAGAGGAGT 3’  5’ CAGTGGCAGTAAGGGCAAG 3’ | 198 |
| ZIC1 | Forward  Reverse | 5’ AAACTGGTTAACCACATCCGC 3’  5’ CTCAAACTCGCACTTGAAGG 3’ | 150 |
| GAPDH | Forward  Reverse | 5’ GAAGGTGAAGGTCGGAGT 3’  5’ GAAGATGGTGATGGGATTTC 3’ | 226 |
